# The Protocol Gap

**DOI:** 10.3390/mps4010012

**Published:** 2021-02-03

**Authors:** Michael G. Weller

**Affiliations:** Federal Institute for Materials Research and Testing (BAM), Richard-Willstaetter-Str. 11, D-12489 Berlin, Germany; michael.weller@bam.de; Tel.: +49-30-8104-1150

**Keywords:** validation, peer review, experiment, documentation, scientific publication, reproducibility crisis, replication crisis, trust, citation, references

## Abstract

Although peer review is considered one of the main pillars of modern science, experimental methods and protocols seem to be not a rigorous subject of this process in many papers. Commercial equipment, test kits, labeling kits, previously published concepts, and standard protocols are often considered to be not worth a detailed description or validation. Even more disturbing is the extremely biased citation behavior in this context, which sometimes leads to surrogate citations to avoid low-impact journals, preprints, or to indicate traditional practices. This article describes some of these surprising habits and suggests some measures to avoid the most unpleasant effects, which in the long term may undermine the credibility of science as a whole.

## 1. Introduction

Recently, an editorial article with the title “The method comes first” was published [1], which opened the discussion whether a new protocol may be published separately after a discovery paper. The fact that a new method was eventually not published in a peer-reviewed journal was criticized. It is very important that these questions are raised. However, when you think twice about this issue, it is evident that this may be only the tip of the iceberg and that some fundamental questions remain, which are briefly discussed here. Finally, a few preliminary recommendations are given, which might show ways for improvement.

## 2. Peer Review and More

It is puzzling to see that a vast number of experimental protocols are not based explicitly on peer-reviewed work. This means that the question of whether a protocol is published before, in, or after a discovery paper is by far not the most relevant one. In addition, it can be questioned whether a traditional peer review is able to elucidate the quality and completeness of a protocol at all. The journal *Organic Syntheses* has followed a fascinating concept starting in 1921 [2]. They only publish protocols, which have “been repeated several times and carefully checked for reproducibility in the laboratory of a member of the Board of Editors.” In most fields, it seems to be nearly impossible to find out for a non-expert which of the many published protocols is reasonably reliable or even “the best” in general or for the specific scientific question.

## 3. The Method Is Not Described at All

The above-mentioned situation might be unintentional or not. In the former case, the authors may have planned to publish a separate method paper, which unfortunately never happened. This is also caused by the difficulty of publishing such papers. Many journals perform something like cherry-picking, which means, in this context, that the discovery paper is considered to be much more attractive, and methods are rarely seen as “exciting” [1]. This may discourage scientists from writing a method paper, even when they planned to do so. Many journals oblige their authors to keep the experimental part as short as possible or to shift the protocols to the Supplementary Materials, even if the method is fundamental to understand the paper or even forms the core of the science discussed. In quite a few journals, the supplement is not subject to peer review, not even on an editorial level. Interestingly, later, after accepting the paper, a “miracle” occurs, and the whole article, including the protocols in the supplement, is acknowledged as peer-reviewed and even validated. Fortunately, today, some journals are specialized in the publication of methods and protocols, such as this journal.

## 4. The Method Was Performed as Usual

Most scientists would agree that this (or an equivalent) statement does not meet the requirements for a scientific publication. However, in an indirect form, similar phrases are used quite frequently, and in most cases, unintentionally. For example, the term “phosphate buffer” does not imply the pH, the concentration, and even the exact composition, not to talk about any additives. Quite often, protocols contain some crucial steps, which are only known in a specific lab. These protocol gaps are sometimes hidden in the use of equipment, compounds, or reagents, which are not described in the literature or are not commercially available. Most reviewers overlook such lacking detail. In plenty of labs, some traditional recipes exist, on which the work of the group is based. Often, they have never been documented explicitly in a refereed journal or even somewhere else, except in an internal lab protocol. In many cases, the authors may consider their method as standard and not worthy to be mentioned. All who ever worked in different labs know that the individual protocols may be highly relevant for success. A similar problem arises with the lack of detail in the citation of antibodies [3] and other very specific biochemical reagents. Although, in theory, a clone number or a protein sequence might avoid such deficiencies, according to some studies, more than 50% of all papers lack such information. It is also surprising that the referees or editors did not raise an objection against such manuscripts, which cannot be reproduced by definition.

## 5. The Method Was Performed as Previously Described (by the Authors)

This statement, combined with one or several citations, appears to be satisfactory for many readers. However, this sometimes affords surprises. First of all, the reference may not lead to the expected protocol, but only to a paper with a similar topic, or also annoying, to another citation, which may lead nowhere. This is even more frustrating if such an irrelevant article was hidden behind an expensive paywall or needed a lot of effort to obtain access to it. Citation cascades may weaken a protocol so much that it is of no value anymore. Besides, most experimental protocols are continuously modified, often in an unobtrusive way in that the authors think these changes do not merit a separate publication. Obviously, after some time, the protocol changed in significant parts, and the citation of the old paper in newer work is not adequate. The recent concept of a “living paper” (similar to [4]) with documented time-stamps might eliminate such problems to some extent.

## 6. The Method Was Performed According to John Doe et al.

This statement is even weaker, particularly when the cited paper was published decades ago. It is not very likely, according to our experience, that any of these protocols are used without some or even major modification. Even when the revision is indicated but not specified in detail, the protocol has to be considered to be incomplete or even irreproducible. The most puzzling fact is the habit that the papers with the highest citation numbers [5] of all times are not necessarily from Nobel prize winners, but describe more or less simple analytical techniques: Although these protocols are outdated in most cases, and therefore, other, quite different methods may have been used instead, they are cited thousandfold. Other similarly essential methods are not cited at all (e.g., because of a commercial source) or only for a short period of time and are subsequently seen as a standard method, not worthy to be mentioned. These extremely biased citation habits show that no generally accepted rules, regarding how methods should be cited, seem to exist.

## 7. The Method Was Performed According to the Manufacturer’s Instructions

This statement has several issues. First of all, most instructions or data sheets of reagents, labeling kits, or test kits contain several options and are formulated in a relatively open way. For example, several buffers may be mentioned, which might be used under a broad range of conditions. This definitely does not mean that the results are the same. In addition, the instructions of a manufacturer are not a peer-reviewed document. These documents often are anonymous and do not show a publication date. Updates are usually not documented, and archives of such documents do not exist in most cases. Furthermore, these instructions can be withdrawn at any time without notice. Finally, some users might modify the protocols somehow without documenting the changes properly. In some cases, literature citations are given in these instruction sheets. However, most often, these citations do not point to a primary publication or a validation of the method and only show the use of the respective commercial product. Sometimes it seems that a commercial supplier even avoids mentioning original papers. Due to copyright issues, the external archiving of these documents is not possible in most cases. Open access publication of such data sheets or instructions by the commercial supplier might be an option for the future.

## 8. Documentation of Complex Devices and Equipment

Complex systems, either commercial or home-made setups, are often not adequately described. Most companies are reluctant to disclose details of their construction, which might be crucial for proper functioning and understanding. Sometimes, even the physical background of such devices is disguised, and original papers are not given. The standard user cannot trace back the source of such a device and is limited to a model number. Even patents, which are considered to be sources of primary information about commercial systems, are not very helpful for this purpose since a patent is not intended to teach the reader unambiguously but, more or less, to confuse and alienate respective competitors. If the company, which delivered the device, goes out of business, details about the systems might be lost irreversibly. In the case of home-built equipment, some groups might avoid describing too much detail in order to keep competitors out of the field.

## 9. Surrogate Citations

Surprisingly, manufacturer’s instructions, textbooks, patents, preprints, Ph.D. theses, or other documents outside of the traditional journals seem to be highly undercited. Today, digital access to some of these records can be monitored, which appears to support this notion. Some authors seem to use a protocol from a source mentioned above and finally cite a somehow related paper from a high-impact journal as a surrogate. Unfortunately, some journals even suggest such practices by discouraging to cite “non-refereed documents” and to prefer “high-quality papers.” Many authors might understand this advice as a request to delete citations of (the real) protocol documents mentioned above and replace them with (surrogate) articles from high-impact journals [5] to improve the chance of the manuscript being accepted.

## 10. Documentation of Software

Most commercial software packages are not open source, and hence, their algorithms are undocumented in most cases. This is a classical black-box application. Even the indication of the version of a software package does not help much, particularly if this specific version is not available anymore: All these results may not be reproducible. This shows the big advantage of open source software, either self-coded or taken from another source—provided that the used version is completely published.

## 11. Paywalls

With the advent of open access publications, their advantages emerged more and more. In the case of experimental protocols, direct and unlimited access to cited documents is quick and not hampered by paywalls. Additionally, the fast and unhindered linkage to other similar papers makes the comparison of similar protocols easier. Even automated tools based on artificial intelligence (AI) might be feasible when full-text processing is admitted.

## 12. Conclusions

Although peer review is considered a central pillar of scientific work, many experimental papers contain significant parts that do not underlie such quality control. Even worse, documentation of large sections is frequently lacking, either due to a commercial context or because the method is considered to be standard.

It seems to be important to remind us of the still unresolved reproducibility crisis, which has been discussed repeatedly, e.g., in [6]. In addition, I would like to point attention to the article series published in *The Lancet* in 2014 with the title “Research: increasing value, reducing waste” [7], which addressed the economic damage caused by poor science, including insufficient reports. Furthermore, it should not be forgotten that unsatisfying protocols not only can damage the career and reputation of the scientists who published them but also the many young, aspiring scientists who relied on their validity.

As it was mentioned in the respective editorial [1], which inspired this article, any results based on undocumented or non-validated methods might put the whole research into question. However, most measures, which are proposed or already implemented already, may not resolve the problem but mainly increase the bureaucratic burden. The forms requested by some journals are excessive, and unequivocal proof for their usefulness is still lacking. In addition, their content cannot be validated independently in most cases.

Hence, I would like to suggest some simple measures, which might improve the situation, without increasing the effort too much:

Authors should always describe the full protocol of their methods used in their paper. In the digital age, at least in the supplement, plenty of space is available. It is nearly impossible to report too much detail. External protocols should only be cited if it is interesting to discuss their context and not as a substitute for a description. It seems to be obvious that protocols should not be hidden behind paywalls. This lesson was ultimately learned during the coronavirus pandemic. Why should this be different when SARS-CoV-2 is defeated?

Referees should not accept papers in which the experiments cannot be reconstructed in all aspects. Citations of general protocols or methods should not be counted as an equivalent of the experimental documentation, only as an acknowledgment of previous work and an indication of a potential external validation. Referees, who have no practical experience in methods used in a manuscript, should refuse to write a review. Only someone with long-term experience with a method can spot the weak points. To assign referees specialized in specific methods might be an option.

Editors should refrain from limiting experimental descriptions either in length or from specific sources. Notably, the implicit pressure to use surrogate citations should be avoided, which is often disguised as an effort for more quality in science. Any reference of a protocol with a DOI should be considered to be acceptable. A preprint with a detailed protocol used in a respective work is definitely preferable to a vague citation of a 50-year-old “high-impact” paper, from which the authors often do not even know the full text.

In the long-term, the complete separation of method documentation and discovery papers might lead to a new era of scientific publication, particularly when the protocols in method papers are published online as “living papers,” including all improvements of a protocol and any changes documented with time-stamps. Furthermore, the benefit of peer review in its present form should again be discussed if large parts of scientific publications unintentionally seem to get along without it. Finally, validation efforts should be bundled in a useful and transparent way, similar to the concept used by *Organic Syntheses*, founded 100 years ago [2]. In any case, all who are involved in the publishing of experimental work should consider: Mind the (protocol) gap!
